# A Comparison between Brachial and Echocardiographic Systolic Time Intervals

**DOI:** 10.1371/journal.pone.0055840

**Published:** 2013-02-07

**Authors:** Ho-Ming Su, Tsung-Hsien Lin, Po-Chao Hsu, Chun-Yuan Chu, Wen-Hsien Lee, Szu-Chia Chen, Chee-Siong Lee, Wen-Chol Voon, Wen-Ter Lai, Sheng-Hsiung Sheu

**Affiliations:** 1 Division of Cardiology, Department of Internal Medicine, Kaohsiung Medical University Hospital, Kaohsiung Medical University, Kaohsiung, Taiwan; 2 Division of Nephrology, Department of Internal Medicine, Kaohsiung Medical University Hospital, Kaohsiung Medical University, Kaohsiung, Taiwan; 3 Department of Internal Medicine, Kaohsiung Municipal Hsiao-Kang Hospital, Kaohsiung Medical University, Kaohsiung, Taiwan; 4 Faculty of Mecicine, College of Medicine, Kaohsiung Medical University, Kaohsiung, Taiwan; Tokai University, Japan

## Abstract

Systolic time interval (STI) is an established noninvasive technique for the assessment of cardiac function. Brachial STIs can be automatically determined by an ankle-brachial index (ABI)-form device. The aims of this study are to evaluate whether the STIs measured from ABI-form device can represent those measured from echocardiography and to compare the diagnostic values of brachial and echocardiographic STIs in the prediction of left ventricular ejection fraction (LVEF) <50%. A total of 849 patients were included in the study. Brachial pre-ejection period (bPEP) and brachial ejection time (bET) were measured using an ABI-form device and pre-ejection period (PEP) and ejection time (ET) were measured from echocardiography. Agreement was assessed by correlation coefficient and Bland-Altman plot. Brachial STIs had a significant correlation with echocardiographic STIs (r = 0.644, *P*<0.001 for bPEP and PEP; r  = 0.850, *P*<0.001 for bET and ET; r = 0.708, *P*<0.001 for bPEP/bET and PEP/ET). The disagreement between brachial and echocardiographic STIs (brachial STIs minus echocardiographic STIs) was 28.55 ms for bPEP and PEP, -4.15 ms for bET and ET and -0.11 for bPEP/bET and PEP/ET. The areas under the curve for bPEP/bET and PEP/ET in the prediction of LVEF <50% were 0.771 and 0.765, respectively. Brachial STIs were good alternatives to STIs obtained from echocardiography and also helpful in prediction of LVEF <50%. Brachial STIs automatically obtained from an ABI-form device may be helpful for evaluation of left ventricular systolic dysfunction.

## Introduction

Systolic time interval (STI) is an established noninvasive technique for the quantitative assessment of cardiac performance and remains valuable for clinical application [1]. Previous studies have shown that STIs have significant diagnostic and prognostic value in heart failure and are adequate for long-term patient follow-up and disease management [2], [3]. Prolonged pre-ejection period (PEP) and shortened ejection time (ET) have been reported to be significantly correlated with decreased left ventricular systolic function [1], [4], [5]. Because heart function impairment usually prolongs PEP and shortens ET, the ratio of PEP to ET may enhance the diagnostic value for the identification of left ventricular systolic dysfunction. A high correlation between PEP/ET and left ventricular ejection fraction (LVEF) has been shown in patients with a wide variety of heart disease [4]. However, STIs are frequently obtained from echocardiography, which may preclude their application in evaluating left ventricular systolic function if echocardiography or adequately trained operators are not available.

A clinical device, ABI-form (Colin VP1000, Komaki, Japan), has been developed to automatically and simultaneously measure blood pressures in both arms and ankles and records pulse waves of the brachial and posterior tibial arteries using an automated oscillometric method. Using this device, we can easily and automatically calculate the brachial pre-ejection period (bPEP) and brachial ejection time (bET) by analyzing the signals of electrocardiogram, phonocardiogram and brachial pressure volume waveform [6]. In our previous studies, the bPEP/bET was reported to have a significant correlation with LVEF and be a useful parameter in prediction of impaired left ventricular systolic function and increased left ventricular mass index (LVMI) [7], [8]. Recently, we have also found the bPEP/bET was an independent predictor for adverse outcomes in patients with chronic renal failure [9], [10] and had a significant impact on the relationship between arterial stiffness and left ventricular hypertrophy (LVH) [11]. However, there has been no study validating the correlation between brachial STIs and STIs obtained from echocardiography. The aims of this study are to evaluate whether the STIs measured from ABI-form device can represent those measured from echocardiography and to compare the diagnostic values of brachial and echocardiographic STIs in the prediction of LVEF <50%.

## Subjects and Methods

### Study Patients and Design

Study subjects were randomly included from a group of patients who arranged for echocardiographic examinations at Kaohsiung Municipal Hsiao-Kang Hospital from April 2010 to October 2011 because of suspicion of coronary artery disease, heart failure, hypertension, abnormal cardiac physical examination, and so on. Patients with significant aortic or mitral valve disease, atrial fibrillation or inadequate image visualization (n = 43) were excluded. We did not include all patients consecutively because bPEP and bET must be measured within 5 min after the completion of an echocardiographic examination. A total of 849 patients (mean age 61.6±13.5 years, 470 males/379 females) were included.

### Ethics Statement

The study protocol was approved by the institutional review board of the Kaohsiung Medical University Hospital (KMUH-IRB-20120157). Informed consents have been obtained in written form from patients and all clinical investigation was conducted according to the principles expressed in the Declaration of Helsinki. The patients gave consent for the publication of the clinical details.

### Evaluation of Cardiac Structure and Function

**Figure 1:**
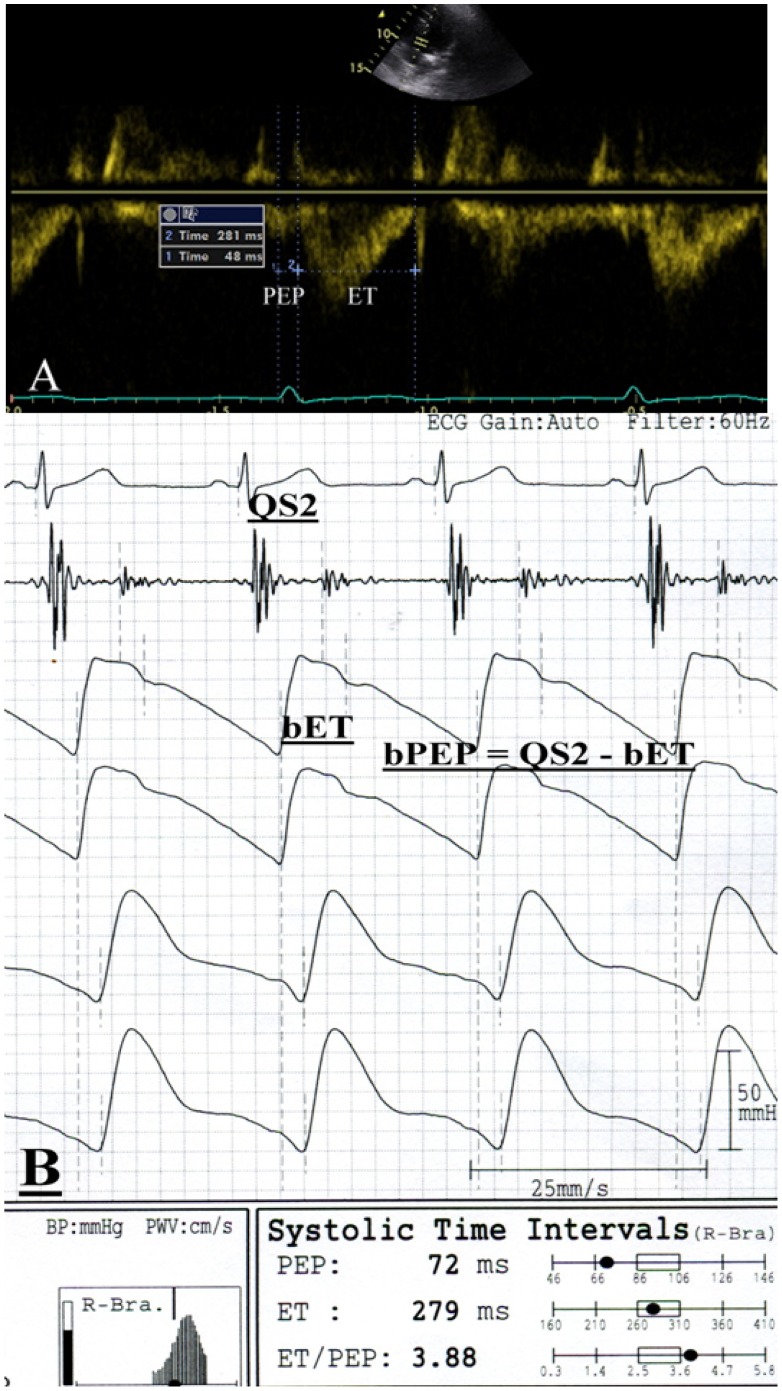
A representative case to illustrate the measurements of pre-ejection period (PEP), ejection time (ET), brachial PEP and brachial ET. In this case, the ET measured from the onset to the end of systolic flow of left ventricular outflow tract (LVOT) was 281 ms and PEP measured from the onset of the QRS complex to the onset of LVOT systolic flow was 48 ms (A). The bET measured from the foot to the dicrotic notch of the brachial pulse volume waveform was 279 ms and QS_2_ measured from the onset of the QRS complex to the first high-frequency vibrations of the aortic component of the second heart sound was 351 ms. The bPEP automatically calculated by QS_2_– bET was 72 ms (B). The bET was slightly shorter than the ET and the bPEP was longer than the PEP.

The echocardiographic examination was performed by one experienced cardiologist with a VIVID 7 (General Electric Medical Systems, Horten, Norway), with the participant respiring quietly in the left decubitus position. The cardiologist was blind to the other data. Two-dimensional and two-dimensionally guided M-mode images were recorded from the standardized views. The Doppler sample volume was placed at the tips of the mitral leaflets to obtain the left ventricular inflow waveforms from the apical 4-chamber view. All sample volumes were positioned with ultrasonic beam alignment to flow. Pulsed tissue Doppler imaging was obtained with the sample volume placed at the lateral corner of the mitral annulus from the apical 4-chamber view. The wall filter settings were adjusted to exclude high-frequency signals and the gain was minimized. The echocardiographic measurements included left ventricular internal diameter in diastole (LVIDd), left ventricular posterior wall thickness in diastole (LVPWTd), interventricular septal wall thickness in diastole (IVSTd), E-wave deceleration time, transmitral E wave velocity (E), transmitral A wave velocity and early diastolic mitral velocity (Ea). Left ventricular systolic function was assessed by LVEF. Left ventricular mass was calculated using Devereux-modified method, i.e. left ventricular mass = 1.04 × [(IVSTd+LVIDd+LVPWTd)^3^– LVIDd^3^] –13.6 g [12]. LVMI was calculated by dividing left ventricular mass by body surface area. LVH was defined as suggested by the the American Society of Echocardiography/European Society of Echocardiography chamber quantification guidelines [13]. Left ventricular relative wall thickness (LVRWT) was calculated as the ratio of 2 × LVPWTd/LVIDd. Cardiac remodeling was defined as LVRWT more than 0.42 without LVH. Concentric LVH was defined as LVMI more than 115 g/m^2^ in men and more than 95 g/m^2^ in women, with LVRWT more than 0.42; eccentric LVH was defined as LVMI more than 115 g/m^2^ in men and more than 95 g/m^2^ in women, with LVRWT less than 0.42. The left atrial volume was measured by the biplane area–length method [13]. Apical 4- and 2-chamber views were obtained to determine the left atrial area and length (from the middle of the plane of the mitral annulus to the posterior wall). The maximal left atrial chamber area and length were measured before mitral valve opening, excluding the left atrial appendage and pulmonary veins. Left atrial volume index was calculated by dividing left atrial volume by body surface area. PEP was measured from the onset of the QRS complex on the electrocardiogram to the onset of systolic flow from the left ventricular outflow tract (LVOT). ET was measured from the onset to the end of LVOT systolic flow (Figure 1A). The PEP and ET were obtained from 3 consecutive beats and then the data were averaged to give the mean value for later analysis. The raw ultrasonic data were recorded and analyzed offline by a cardiologist, blinded to the other data, using EchoPAC software (GE Medical Systems).

### Assessment of bPEP and bET

The bPEP and bET were measured by using an ABI-form device, which automatically and simultaneously measures blood pressures in both arms and ankles using an oscillometric method [6], [14]–[16]. The bET were automatically measured from the foot to the dicrotic notch (equivalent to the incisura on the downstroke of the aortic pressure wave contour produced by the closure of aortic valve) of the pulse volume waveform. Total electromechanical systolic interval (QS_2_) was measured from the onset of the QRS complex on the electrocardiogram to the first high-frequency vibrations of the aortic component of the second heart sound on the phonocardiogram. The bPEP was also automatically calculated by subtracting the bET from the QS_2_ (Figure 1B). The validation of this automatic device and its reproducibility have been previously published [6]. The mean percentage errors for bPEP, bET and bPEP/bET measurement (3.6±3.6%, 2.0±1.5% and 4.2±4.4%, respectively) have been reported in our previous study [7].

### Collection of Demographic and Medical Data

Demographic and medical data including age, gender, smoking history and comorbid conditions were obtained from medical records or interviews with patients. The body mass index was calculated as the ratio of weight in kilograms divided by square of height in meters.

### Statistical Analysis

Statistical analysis was performed using SPSS 15.0 for windows (SPSS Inc. Chicago, USA). Data are expressed as percentages or mean ± standard deviation. The differences in items between brachial and echocardiographic STIs were checked by paired Student’s *t*-test. The relationship between two continuous variables was assessed using a bivariate correlation method (Pearson’s correlation). Bland-Altman plots were used to assess the agreements between brachial and echocardiographic STIs. The regression of the average and the difference between brachial and echocardiographic STIs (brachial STIs minus echocardiographic STIs) was analyzed. Receiver operating characteristic (ROC) curve was constructed for the prediction of LVEF <50%. A difference was considered significant if the *P* value was less than 0.05.

## Results

There were 849 patients included in this study. The clinical characteristics of these patients were listed in Table 1. The mean age was 61.6±13.5 years and male sex compromised 55.4% of the patients. More than a quarter of our patients (28.9%) were diabetic and 71.6% of patients had hypertension. Pre-existed and documented coronary artery and cerebrovascular diseases were noted in 19.1% and 5.9% of patients, respectively.

**Table 1:** Clinical characteristics of study patients.

Characteristics	All patients (n = 849)
Age (year)	61.6±13.5
Male gender (%)	55.4
Smoking history (%)	14.4
Diabetes mellitus (%)	28.9
Hypertension (%)	71.6
Coronary artery disease (%)	19.1
Cerebrovascular disease (%)	5.9
Systolic blood pressure (mmHg)	135.8±20.8
Diastolic blood pressure (mmHg)	76.9±11.8
Pulse pressure (mmHg)	58.9±14.2
Heart rate (beats/min)	69.7±12.1
Body mass index (kg/m^2^)	26.2±3.9
bPEP (ms)	101.6±17.9
bET (ms)	286.0±33.3
bPEP/bET	0.36±0.09
Echocardiographic data	
PEP (ms)	73.1±16.4
ET (ms)	290.2±34.6
PEP/ET	0.26±0.08
LAVI (ml/m^2^)	34.1±14.5
LV relative wall thickness	0.39±0.10
LVMI (g/m^2^)	137.2±45.4
LV geometry	
non-LVH	18.3
concentric remodeling	8.2
eccentric LVH	48.2
concentric LVH	25.3
LVEF (%)	63.5±12.1
E (cm/s)	70.7±21.7
A (cm/s)	79.8±27.2
E/A	0.95±0.42
Ea (cm/s)	8.6±3.2
E/Ea	9.4±4.8
E-wave deceleration time (ms)	207.5±65.2

Abbreviations. bPEP, brachial pre-ejection period; bET, brachial ejection time; PEP, pre-ejection period; ET, ejection time; LAVI, left atrial volume index; LV, left ventricular; LVMI, left ventricular mass index; LVH, left ventricular hypertrophy; LVEF, left ventricular ejection fraction; E, transmitral E wave velocity; A, transmitral A wave velocity; Ea, early diastolic mitral velocity.

### Comparison between Brachial and Echocardiographic STIs

The values of bPEP and PEP were 101.6±17.9 and 73.1±16.4 ms (*P*<0.001), respectively and those of bET and ET were 286.0±33.3 and 290.2±34.6 ms (*P*<0.001), respectively. Besides, the values of bPEP/bET and PEP/ET were 0.36±0.09 and 0.26±0.08 (*P*<0.001), respectively. The measurement time of bPEP/bET derived from 20 consecutive patients, starting from placement to removal of cuffs and electrodes, was 4.74±0.35 minutes and the cost was around $17 for each person.

**Figure 2:**
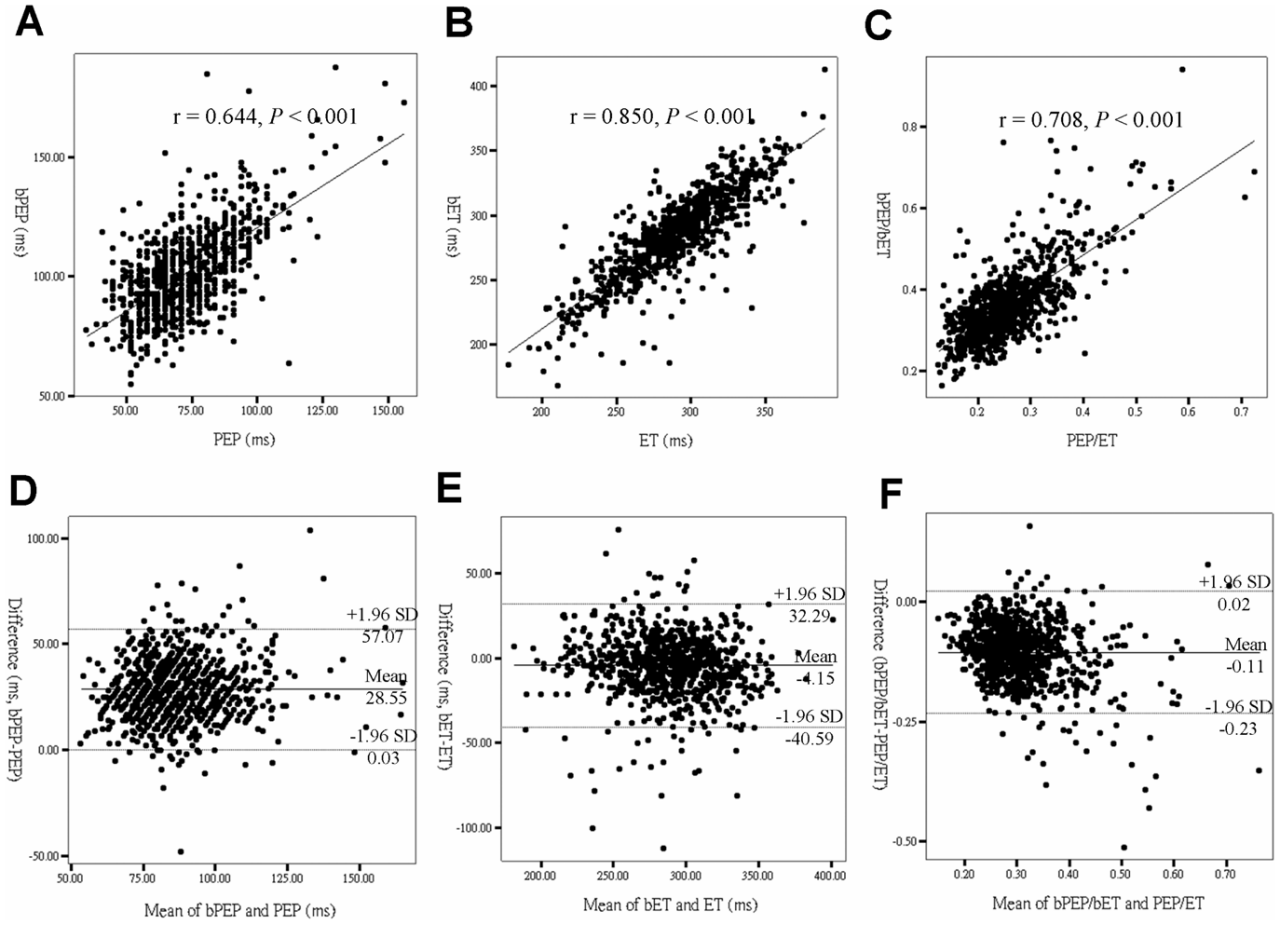
Regression plots between pre-ejection period (PEP) and brachial PEP (bPEP) (A), ejection time (ET) and brachial ET (bET) (B) and PEP/ET and bPEP/bET (C) and Bland-Altman plots of PEP and bPEP (D), ET and bET (E) and PEP/ET and bPEP/bET (F) in all patients.

Figure 2 shows the regression plots between bPEP and PEP (r = 0.644, *P*<0.001) (A), bET and ET (r = 0.850, *P*<0.001) (B) and bPEP/bET and PEP/ET (r = 0.708, *P*<0.001) (C). To assess the agreement between brachial and echocardiographic STIs, Bland-Altman plots are produced (Figure 2). Mean bPEP overestimates PEP on average (mean difference, 28.55 ms) and the 95% limit of agreement is 0.03 to 57.07 ms (Figure 2D). The bET underestimates ET (mean difference, -4.15 ms) and the 95% limit of agreement is 32.29 to -40.59 ms (Figure 2E). In addition, bPEP/bET overestimates PEP/ET (mean difference, 0.11) and the 95%limit of agreement is 0.02 to -0.23 (Figure 2F).

### Comparisons of Brachial and Echocardiographic STIs in Prediction of LVEF <50%

**Figure 3:**
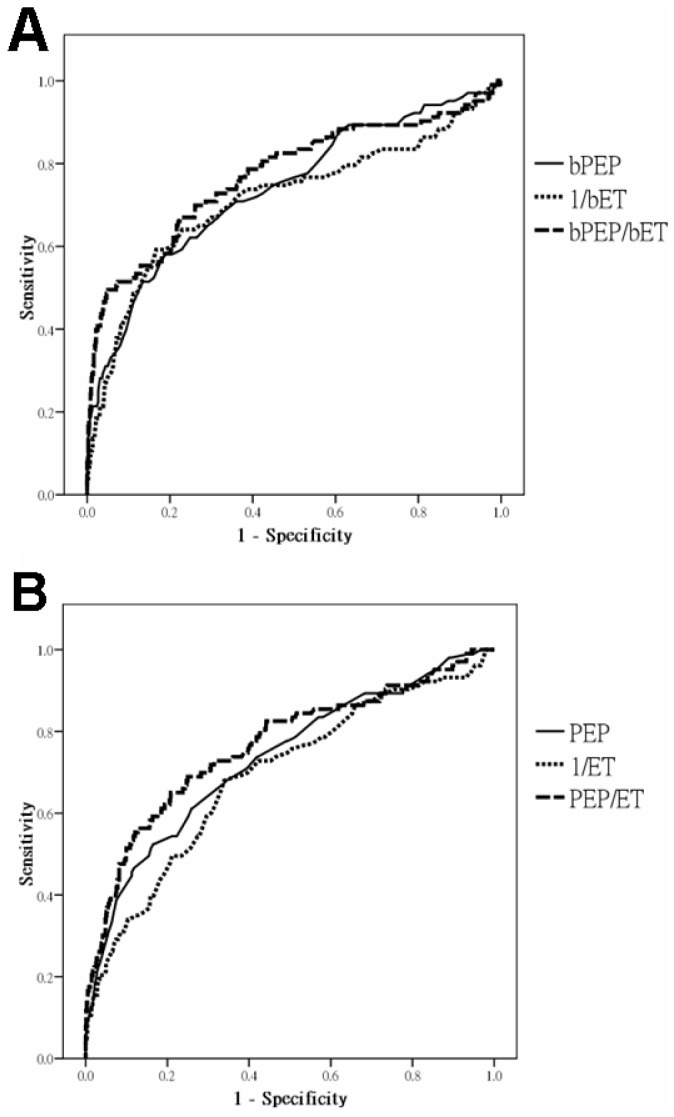
ROC curves for brachial pre-ejection period (bPEP), 1/brachial ejection time (bET) and bPEP/bET (A) and pre-ejection period (PEP), 1/ejection time (ET) and PEP/ET from echocardiography (B) in the prediction of left ventricular ejection fraction <50%.

The prevalence of LVEF <50% is 12.2%. The ROC curves for bPEP, 1/bET and bPEP/bET (A) and PEP, 1/ET and PEP/ET (B) in the prediction of LVEF <50% are shown in Figure 3. The areas under the curve (AUC) for bPEP, 1/bET and bPEP/bET in the prediction of LVEF <50% are 0.738, 0.715 and 0.771, respectively. In addition, the AUC for PEP, ET and PEP/ET in the prediction of LVEF <50% are 0.732, 0.691 and 0.765, respectively. In addition, we also calculated the statistical values of bPEP>105.5 ms, bET <277.5 ms, bPEP/bET >0.38, PEP>77.5 ms, ET <280.5 ms and PEP/ET >0.27 in prediction of LVEF <50% (Table 2).

**Table 2:** The statistical values of bPEP, bET, bPEP/bET, PEP, ET andPEP/ET in prediction of LVEF <50%.

	Sensitivity (%)	Specificity (%)	Accuracy (%)
bPEP>105.5 ms	68.9	66.5	66.8
bET<277.5 ms	68.9	67.3	67.5
bPEP/bET >0.38	70.9	71.0	71.0
PEP>77.5 ms	67.0	66.6	66.7
ET<280.5 ms	67.0	66.4	66.4
PEP/ET >0.27	69.9	69.8	69.8

LVEF, left ventricular ejection fraction; bPEP, brachial pre-ejection period; bET, brachial ejection time; PEP, pre-ejection period; ET, ejection time.

Because heart rate, a history of coronary artery disease and left ventricular systolic function might influence the STIs, we also further performed subgroup analyses of patients divided by median of heart rate (68 beats/min), a history of coronary artery disease and LVEF (50%). In a univariate correlation analysis, heart rate had a significant correlation with bPEP (r = −0.075, *P* = 0.029), bET (r = −0.719, *P*<0.001), ET (r = −0.681, *P*<0.001), bPEP/bET (r = 0.329, *P*<0.001) and PEP/ET (r = 0.293, *P*<0.001), but not achieving significance with PEP (r = −0.027, *P* = 0.438). Table 3 shows the correlation between brachial and echocardiographic STIs and the AUC of these STIs in the prediction of LVEF <50% in subgroup analysis. The AUC for bPEP/bET and PEP/ET in the prediction of LVEF <50% are 0.784 and 0.816 in patients with heart rate>median and 0.718 and 0.690 in patients with heart rate≤median. In addition, the AUC for bPEP/bET and PEP/ET in the prediction of LVEF <50% are 0.670 and 0.624 in patients with coronary artery disease and 0.807 and 0.835 in patients without coronary artery disease. The clinical characteristics of these subgroup patients are shown in Table 4.

**Table 3:** Correlation between brachial and echocardiographic STIs and AUC of these STIs in the prediction of LVEF <50% in subgroup analysis.

Parameter	HR>median (68 beats/min) (n = 413)		HR<median (68 beats/min) (n = 436)		CAD (+) (n = 162)		CAD (−) (n = 687)		LVEF <50% (n = 103)		LVEF >50% (n = 746)		
	r	*P*	r	*P*	r	*P*	r	*P*	r	*P*	r	*P*	
bPEP and PEP (ms)	0.657	<0.001	0.630	<0.001	0.512	<0.001	0.751	<0.001	0.637	<0.001	0.589	<0.001	
bET and ET (ms)	0.799	<0.001	0.780	<0.001	0.828	<0.001	0.841	<0.001	0.871	<0.001	0.834	<0.001	
bPEP/bET and PEP/ET	0.724	<0.001	0.667	<0.001	0.585	<0.001	0.782	<0.001	0.686	<0.001	0.631	<0.001	
	AUC for LVEF <50%				AUC for LVEF <50%								
bPEP/bET	0.784		0.718		0.670		0.807						
PEP/ET	0.816		0.690		0.624		0.835						

Abbreviations. bPEP, brachial pre-ejection period; bET, brachial ejection time; AUC, area under curve; HR, heart rate; CAD, coronary artery disease; LVEF, left ventricular ejection fraction.

**Table 4:** Clinical characteristics of subgroup patients.

Characteristics	HR>median (68 beats/min) (n = 413)	HR<median (68 beats/min) (n = 436)	CAD (+) (n = 162)	CAD (−) (n = 687)	LVEF <50% (n = 103)	LVEF >50% (n = 746)
Age (year)	60.5±14.2	62.6±12.7*	63.7±12.0	61.1±13.7*	63.9±14.8	61.3±13.2
Male gender (%)	56.4	54.4	80.2	49.5**	68.0	53.7*
Smoking history (%)	14.5	14.3	19.8	13.1*	20.4	13.6
Diabetes mellitus (%)	32.7	25.2*	42.0	25.8**	35.0	28.0
Hypertension (%)	72.1	71.1	63.4	73.5*	56.3	73.6**
CAD (%)	15.0	22.9*	100	0**	47.6	15.1**
Cerebrovascular disease (%)	5.1	6.7	6.8	5.7	7.8	5.6
Systolic blood pressure (mmHg)	136.5±20.3	135.2±21.2	130.1±19.7	137.2±20.8**	128.7±21.6	136.8±20.5**
Diastolic blood pressure (mmHg)	78.9±11.8	75.0±11.4**	73.4±9.9	77.7±12.0**	74.6±13.2	77.2±11.6*
Pulse pressure (mmHg)	57.5±14.0	60.2±14.3*	56.7±1.45	59.4±14.1*	54.0±14.5	59.6±14.1**
HR (beats/min)	79.2±9.5	60.6±5.4**	66.2±11.0	70.5±12.2**	76.0±17.2	68.8±11.0**
Body mass index (kg/m^2^)	26.3±4.3	26.1±3.6	26.4±3.4	26.1±4.1	25.6±4.3	26.3±3.9
bPEP (ms)	99.8±19.5	103.4±16.2*	102.4±16.9	101.4±18.2	118.0±25.2	99.4±15.4**
bET (ms)	266.5±28.9	304.5±25.7**	290.7±32.2	284.9±33.4	261.1±41.5	289.4±30.5**
bPEP/bET	0.38±0.10	0.34±0.07**	0.36±0.09	0.36±0.09	0.47±0.14	0.35±0.07**
Echocardiographic data						
PEP (ms)	71.8±16.5	74.2±16.3*	72.4±16.2	73.2±16.5	87.4±21.8	71.1±14.5**
ET (ms)	271.9±30.1	307.5±29.2**	296.9±34.2	288.6±34.5*	268.2±39.7	293.2±32.7**
PEP/ET	0.27±0.08	0.25±0.07**	0.25±0.07	0.26±0.08	0.34±0.11	0.25±0.06**
LAVI (ml/m^2^)	33.3±15.2	35.0±13.7	37.0±15.2	33.5±14.3*	46.7±18.7	32.4±13.0**
LV relative wall thickness	10.0±1.9	9.9±1.7	9.9±1.7	10.0±1.8	9.6±1.9	10.0±1.8*
LVMI (g/m^2^)	134.8±49.7	139.5±40.9	142.8±44.5	135.9±45.6	182.1±58.4	131.0±39.6**
LV geometry						
non-LVH	18.2	18.3	21.0	17.6	7.8	19.8*
concentric remodeling	12.8	3.9**	8.6	9.3*	0	9.4*
eccentric LVH	43.3	52.8*	54.3	46.7	82.5	43.3**
concentric LVH	25.7	25.0	21.0	26.3	9.7	27.6**
LVEF (%)	62.2±12.7	64.8±11.3*	57.4±13.3	65.0±11.3**	38.4±8.4	67.0±7.5**
E (cm/s)	69.8±21.8	71.6±21.5	69.1±20.3	71.1±22.0	71.1±27.0	70.7±20.9
A (cm/s)	82.0±22.5	77.8±21.7*	77.7±19.5	80.3±22.8	74.1±23.9	80.6±21.9*
E/A	0.9±0.4	1.0±0.4*	1.0±0.4	1.0±0.4	1.1±0.7	0.9±0.4*
Ea (cm/s)	8.6±3.4	8.6±3.1	7.5±2.7	8.8±3.3**	5.7±2.5	9.0±3.1**
E/Ea	9.4±5.1	9.5±4.6	10.5±5.3	9.2±4.7*	14.2±6.7	8.8±4.2**
E-wave deceleration time (ms)	200.5±62.7	214.2±66.8*	207.9±66.5	207.4±64.9	187.5±75.3	210.3±63.3*

Abbreviations. HR, heart rate; CAD, coronary artery disease; LVEF, left ventricular ejection fraction; bPEP, brachial pre-ejection period; bET, brachial ejection time; PEP, pre-ejection period; ET, ejection time; LAVI, left atrial volume index; LV, left ventricular; LVMI, left ventricular mass index; LVH, left ventricular hypertrophy; E, transmitral E wave velocity; A, transmitral A wave velocity; Ea, early diastolic mitral velocity.

* *P*<0.05,

** *P*<0.001 compared to counterpart group.

## Discussion

As a tool for left ventricular systolic function and to determine the efficacy of novel treatments to improve cardiac dysfunction, having an accurate and validated STIs is extremely important in the clinical practices and epidemiologic studies. In the present study, we evaluate the correlation between brachial STIs measured from the ABI-form device and STIs measured from echocardiography. We found that brachial STIs were good alternatives to STIs obtained from echocardiography and also helpful in identification of LVEF <50%. Hence, brachial STIs were cheap, useful, automatically and easily obtained parameters in evaluation of left ventricular systolic function, so they might be helpful for large-scale screening to identify patients with impaired left ventricular systolic function.

Cybulski et al. [17] had investigated a comparison between the automatized impedance cardiography (ICG) and pulse-wave Doppler echocardiography methods for measurements of STIs in 9 healthy male subjects with a mean age of 24.9 years. The PEP estimated by ICG was 22 ms longer than that determined by echocardiography (*P*<0.001) but there was no difference between ET determined by the two methods (*P*>0.9). In our study, the bPEP overestimates PEP (mean difference, 28.55 ms) and the bET underestimates ET (mean difference, -4.15 ms). In this study, PEP+ET was measured from the onset of the QRS complex on the electrocardiogram to the end of LVOT systolic flow. However, bPEP+bET was equal to QS_2_, the onset of the QRS complex on the electrocardiogram to the first high-frequency vibrations of the aortic component of the second heart sound on the phonocardiogram. Hence, the bPEP+bET should be very close to the time interval measured from the onset of the QRS complex on the electrocardiogram to the end of “aortic” systolic flow. Because the end of aortic systolic flow was preceded by the end of LVOT systolic flow, the bPEP+bET was inherently longer than the PEP+ET. Furthermore, the bET was slightly shorter than the ET, so the bPEP was certainly longer than the PEP in the present study. In addition, the ET was measured from the onset to the end of LVOT systolic flow, but the bET was measured from the foot to the dicrotic notch of the pulse volume waveform. The different measurement methods and sites might be the possible explanation for the small difference between bET and ET. Although brachial and echocardiographic STIs were not the same, when compared to their counterparts, these parameters were all helpful and comparable in the prediction of impaired left ventricular systolic function.

In the physiologic range, the PEP is less influenced by heart rate than ET [3], [7]. Our study showed the similar findings that bET and ET were much influenced by heart rate than bPEP and PEP. Decreased heart rate is associated with increased ET and then decreased the ratio of PEP to ET, resulting in a phenomenon of “pseudonormalization” of PEP/ET. In this study, we found that the AUC for bPEP/bET and PEP/ET in prediction of LVEF <50% was lower in patients with slow heart rate than in those with rapid heart rate. This finding implied that the predictive values of left ventricular systolic dysfunction by bPEP/bET and PEP/ET would be influenced by heart rate and might be less accurate in patients with slow heart rate.

Paiva et al. [18] had evaluated the feasibility of using heart sound and electrocardiogram to measure the STIs and found that when compared to those measured from echocardiography, absolute estimation errors of PEP and ET were 7.66 and 11.39 ms in a healthy population and 11.86 and 17.51 ms in the subjects with different cardiovascular disease, respectively. In our study, the predictive values of left ventricular systolic dysfunction by bPEP/bET and PEP/ET were better in patients without coronary artery disease than in those with coronary artery disease. The possible explanation was that patients with coronary artery disease might have a localized infarction, scattered fibrosis, or ischemic noncontractile myocardium, resulting in impaired left ventricular systolic function [19], [20], but the STIs were mainly used in evaluating global heart function [1]. Therefore, bPEP/bET and PEP/ET seemed to be less reliable in identification of patients with LVEF <50% in patients with coronary artery disease.

There are several limitations to this study. The majority of our patients were treated chronically with antihypertensive medications. For ethical reasons, we did not withdraw these medications. Hence, we could not exclude the influence of antihypertensive agents on the present findings. Besides, because the measurements of bPEP, bET, PEP and ET during atrial fibrillation were difficult because of beat-to-beat variation, we excluded patients with atrial fibrillation. Hence, our results could not be applied in these patients.

In conclusion, the present study demonstrated that brachial STIs were good alternatives to STIs obtained from echocardiography. Although brachial and echocardiographic STIs were not the same, brachial STIs for identifying left ventricular systolic dysfunction were as good as the echocardiographic STIs. Hence, brachial STIs automatically obtained from an ABI-form device may be helpful for identification of patients with left ventricular systolic dysfunction.
